# Metabolic Modulation of Type 2 Diabetes Mellitus by 1-Deoxynojirimycin: A Multifaceted Approach

**DOI:** 10.3390/antiox15050585

**Published:** 2026-05-05

**Authors:** Yingying Zhang, Pravin Ojha, Xia Tang, Liangfu Zhou, Yasai Sun, Qinghai Sheng

**Affiliations:** 1 College of Food Science & Technology, Hebei Agricultural University, Baoding 071000, China; 20241070032@pgs.hebau.edu.cn (Y.Z.); projha84@narc.gov.np (P.O.); ndsptx@hebau.edu.cn (X.T.); zlf2022@hebau.edu.cn (L.Z.); sunyasai@hebau.edu.cn (Y.S.); 2 Chengde Academy of Agricultural and Forestry Science, Chengde 067000, China; 3 National Food Research Centre, Nepal Agricultural Research Council, Lalitpur 44700, Nepal; 4 Hebei Province Key Laboratory of Dietary Component Interaction and Precision Nutrition, Baoding 071000, China

**Keywords:** DNJ, adipose tissues, insulin resistance, intestinal microbiota, network pharmacology

## Abstract

Type 2 diabetes mellitus (T2DM) represents a significant global health burden. The natural alkaloid, 1-Deoxynojirimycin (DNJ), abundant in mulberry (*Morus alba* L.), offers a promising bioactive approach to its early management. This review comprehensively summarises the multifaceted roles of DNJ in modulating the core pathophysiological dysfunctions of T2DM, including impaired glucose and lipid metabolism, insulin resistance (IR), and dysbiosis of gut microbiota. Specifically, DNJ exerts its therapeutic effects by regulating various pathways involved in glucose and lipid metabolism (e.g., phosphatidylinositol 3-kinase (PI3K)-protein kinase B (AKT) and AMP-activated protein kinase (AMPK) pathways), enhancing insulin sensitivity, modulating the gut microbiota, and upregulating transporter proteins. We highlight emerging methodologies, such as network pharmacology, which underscore the pivotal role of the PI3K/AKT and AMPK signalling pathways as primary targets of DNJ in T2DM management. Although this review elucidates multifaceted mechanisms of DNJ in T2DM management, it also identifies critical research gaps, particularly concerning its effects on pancreatic cells, obesity-related T2DM, and mitochondrial energy metabolism. Further investigation in these areas is crucial for fully understanding DNJ’s preventive and therapeutic potential and for the development of related functional foods.

## 1. Introduction

Diabetes mellitus (DM) is a major global health concern. DM is primarily classified as type 1 or type 2, with more than 90% of patients diagnosed with type 2 diabetes mellitus (T2DM). T2DM is considered the eighth leading cause of disease burden globally and is estimated to become the second-most common disease by 2050 [1,2]. The pathophysiology of T2DM is highly complex and encompasses multiple interrelated mechanisms, including insulin resistance (IR), pancreatic *β*-cell dysfunction, dysregulated lipid metabolism, attenuated incretin effect, gut microbiota dysbiosis, elevated oxidative stress, inflammation, and mitochondrial dysfunction [3,4]. A range of antidiabetic medications is currently available; however, long-term use is often associated with adverse effects, such as gastrointestinal bloating, diarrhoea, and abnormal bowel movements [5,6,7]. Consequently, there is growing interest in identifying natural product-derived compounds with improved safety profiles and minimal side effects. 1-Deoxynojirimycin (DNJ) is a natural compound with minimal side effects and has proven its potential in the management of diabetes.

DNJ is an alkaloid that is abundant in mulberry (*Morus alba* L.) leaves and is found in certain microorganisms (e.g., *Bacillus* spp.) and insects (e.g., *Bombyx mori*) [8,9]. It has been extensively studied for its ability to lower blood sugar [10,11,12,13] (Table 1). This functionality stems from its unique molecular structure, which features a six-membered piperidine ring centred on a nitrogen atom, with hydroxyl groups whose number, position, and stereochemical orientation closely resemble those of glucose. This structural mimicry enables DNJ to act as a highly effective glucose analogue and potent inhibitor of α-glucosidase [14]. Although DNJ is primarily recognised as an α-glucosidase inhibitor that ameliorates hyperglycaemia, it is also an antioxidant that clears free radicals and enhances antioxidant activity [15,16]. In addition, it has beneficial effects in mitigating various chronic conditions, including obesity, cardiovascular disease, inflammation, and cancer [12,17].

While the molecular structure and hypoglycaemic activity of DNJ have been extensively researched [38,39,40], a systematic review focusing on its core regulatory mechanisms and therapeutic potential in T2DM is lacking. This review examines the pathophysiological dysfunctions of T2DM and the regulatory effects of DNJ, specifically on glucose metabolism, lipid metabolism, IR, gut microbiota, and network pharmacology. Furthermore, we address knowledge gaps regarding the effects of DNJ on multiple facets of T2DM, aiming to provide novel insights and directions for the prevention and treatment of T2DM through in-depth pharmacological studies on DNJ.

## 2. Methods

A comprehensive literature search was conducted across multiple databases, including X-MOL, ScienceDirect, PubMed, Google Scholar, and Web of Science. Additionally, data from the International Diabetes Federation were used to obtain the latest diabetes statistics. This review focuses on five key areas: glucose metabolism and lipid metabolism disorders, IR, intestinal microbiota imbalance, and the application of network pharmacology in DNJ regulation in T2DM. The preliminary search used keywords including ‘1-deoxynojirimycin or DNJ,’ ‘type 2 diabetes or T2DM,’ ‘glucose metabolism,’ ‘lipid metabolism,’ ‘insulin resistance,’ ‘gut microbiota or intestinal microbiota,’ and ‘network pharmacology.’ However, the search criteria were systematically adjusted to align with the scope of the study. References were managed using EndNote 21 to ensure organisation and avoid duplication.

Initially, 328 papers were selected for review. Screening was performed based on relevance and inclusion criteria, limited to English-language journals published after 2000. A total of 117 papers were cited, with over 72% of them published after 2020. However, owing to limitations in access, the review may not have covered non-English publications and grey literature; backward citation tracking and repeated screening of earlier literature were employed to minimise potential bias.

## 3. The Alterations in Glucose Metabolism in T2DM and the Role of DNJ

Glucose homeostasis involves complex interactions between absorption from the gut, transport into cells, and subsequent metabolic processing. It is necessary to understand the mechanism by which DNJ modulates glucose uptake, cellular transport, and various metabolic pathways.

### 3.1. Glucose Absorption

Glucose is the major source of monosaccharide energy in the human body. After consumption, α-amylase and α-glucosidase break down carbohydrates into simple sugars, leading to an increase in blood glucose levels. Inhibiting these enzymes effectively reduces glucose release and absorption in the small intestine [41,42]. First, DNJ, which chemically resembles glucose, competitively inhibits intestinal α-glucosidase activity, thereby limiting the supply of glucose substrates available for absorption and reducing the breakdown of disaccharides and polysaccharides into glucose [43]. Although the efficiency of DNJ against α-amylase is comparatively low, its inhibitory properties can be augmented by conjugating it with sugar moieties or by combining it with other natural inhibitors [44,45].

**Figure 1:**
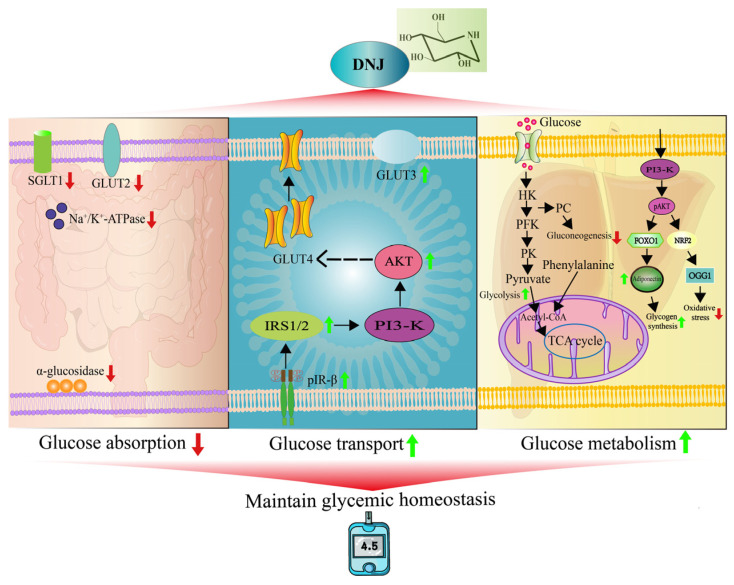
Mechanisms of DNJ in regulating glucose metabolism in T2DM. DNJ mainly ameliorates hyperglycaemia in T2DM through intestinal α-glucosidase inhibition, hepatic glycolytic activation/gluconeogenic suppression, and GLUT4 translocation-mediated glucose uptake in cells. Note: Black arrows indicate upstream and downstream cascade reactions; red arrows indicate a decrease in protein expression levels; green arrows indicate an increase in protein expression levels.

Beyond α-glucosidase inhibition, glucose absorption in the small intestine is primarily mediated by sodium–glucose cotransporter 1 (SGLT1). Therefore, DNJ may reduce intestinal glucose absorption by downregulating SGLT1 expression and/or inhibiting SGLT1 activity [20]. DNJ downregulates the mRNA and protein expression of SGLT1, glucose transporter 2 (GLUT2), and Na^+^/K^+^ adenosine triphosphatease (ATPase) in the intestine, thereby inhibiting intestinal glucose absorption [12]. Although DNJ is known to downregulate SGLT1 expression, the precise molecular mechanism by which it directly inhibits SGLT1 activity, independent of this transcriptional effect, remains unclear and represents a critical gap in our understanding of its glucose absorption-modulating properties. The regulatory role of DNJ in these combined mechanisms can effectively improve blood glucose levels in T2DM patients (Figure 1).

### 3.2. Glucose Transport

Maintaining glucose homeostasis is central to metabolic health and is primarily dependent on the regulation of glucose transport by the insulin signalling pathway. The classic insulin-PI3K-phosphorylated AKT (pAKT) signalling pathway plays a pivotal role in this process. After insulin binds to its receptor substrate, it activates the intrinsic tyrosine kinase domain, leading to the phosphorylation of tyrosine residues on the insulin receptor substrate (IRS). The phosphorylated IRS protein interacts with the regulatory subunit p85 to activate PI3K. Activation of PI3K leads to the phosphorylation of AKT at Ser473, a process crucial for regulating GLUT4 translocation to the cell membrane, thereby enhancing cellular glucose uptake and transport [46]. Dysregulation of this pathway is pivotal in the development and progression of IR and T2DM.

By mimicking insulin, DNJ promotes cellular glucose uptake by upregulating key proteins such as insulin receptor *β* (IR-*β*) phosphorylation, IRS-1, PI3K, and AKT [18,47]. This action enhances GLUT4 translocation to the plasma membrane, thereby alleviating hyperglycaemia by increasing glucose clearance from the bloodstream into cells [48] (Figure 1). Furthermore, DNJ’s influence extends to other glucose transporters; it increases GLUT3 mRNA levels in insulin-resistant cells, which is notable given GLUT3’s primary role as a glucose transporter in neurons [49]. This suggests a potentially broader impact on glucose uptake in various tissues, including the nervous system, beyond the classic insulin-dependent pathways. Increased expression of both directly promotes transmembrane glucose transport.

### 3.3. Glucose Metabolism

Glucose metabolism is a complex and coordinated process. It primarily comprises glycolysis, the tricarboxylic acid (TCA) cycle, the pentose phosphate pathway, glycogen synthesis and breakdown, and gluconeogenesis. As the core pathway for initial glucose utilisation, glycolysis is tightly regulated by hexokinase (HK), phosphofructokinase (PFK), and pyruvate kinase (PK). Notably, DNJ treatment of HFD combined with streptozotocin-induced diabetic mice may delay the onset of diabetes through the methylation levels of CpG sites in the insulin-associated HK1 gene, showing a positive correlation with age and disease incidence, and has become a potential biomarker for diabetes risk. After 90 days, HK, PFK, and PK expression were significantly upregulated in the liver [50,51]. This enhances glucose oxidation, converts glucose into pyruvate, and generates ATP, thereby accelerating hepatic glucose metabolism. This enhanced glycolysis directly impacts the metabolic fate of pyruvate, which, in turn, influences the efficiency of energy utilisation.

The metabolic fate of pyruvate, the final product of glycolysis, directly influences energy utilisation efficiency. A portion of pyruvate enters the mitochondria for complete oxidation via the TCA cycle to generate energy, whereas the remainder is either carboxylated to oxaloacetate by pyruvate carboxylase (PC) or reduced to lactate under hypoxic conditions. Normal functioning of the TCA cycle is crucial for maintaining energy balance and is a common hub for the metabolism of different nutrients. However, in T2DM patients, widespread mitochondrial dysfunction in the skeletal muscle and liver impairs pyruvate conversion to acetyl-CoA and reduces TCA cycle efficiency. This not only diminishes ATP production but also leads to the accumulation of lipid intermediates, thereby disrupting insulin signalling and promoting IR [52]. Furthermore, DNJ facilitates energy production via the TCA cycle in the absence of glucose. DNJ promotes the conversion of phenylalanine to acetyl-CoA [24]. This shifts carbohydrate metabolism towards glycolysis rather than gluconeogenesis (Figure 1).

DNJ modulates the pentose phosphate pathway, which is crucial for nicotinamide adenine dinucleotide phosphate hydrogen (NADPH) production and redox balance. Impaired metabolic flux in this pathway diminishes NADPH production and elevates oxidative stress levels, a chronic condition that critically disrupts insulin signalling and *β*-cell function [53]. According to recent findings [54], 5 μmol/L DNJ stimulates nuclear factor erythroid 2-related factor 2 (NRF2) signalling to activate the AKT/NRF2/8-oxoguanine DNA glycosylase 1 (OGG1) pathway, enhancing the expression of the oxidative DNA damage repair protein, OGG1. This reduces high glucose-induced oxidative stress and accelerates glucose metabolism via the pentose phosphate pathway.

DNJ has been shown to inhibit gluconeogenesis, which is crucial for maintaining stable blood glucose levels during fasting. The key step involves the transport of oxaloacetate across the mitochondrial membrane as malate. This process is driven by glycerol and non-esterified fatty acid-derived acetyl-CoA, which activates the PC. This enzyme stimulates the carboxylation of pyruvate, thereby promoting increased fasting glucose levels. PC is the rate-limiting enzyme in gluconeogenesis and regulates the overall speed of the pathway. In patients with diabetes, excessive hepatic glucose production via gluconeogenesis is the primary driver of elevated blood glucose levels [55]. Combined treatment with DNJ and polysaccharides extracted from mulberry leaves significantly reduced PC protein levels in diabetic mice, indicating diminished gluconeogenic activity and inhibition of the conversion of glucose-6-phosphate to free glucose [51].

Glycogen synthesis and breakdown are crucial components of carbohydrate metabolism, a process that is regulated by hormones and enzymes. Insulin primarily restores glycogen synthase activity by inhibiting adenylate cyclase and phosphodiesterase and promoting dephosphorylation. This process subsequently inhibits the activity of forkhead box protein O1 (FOXO1) [56,57] and gluconeogenic enzymes, while increasing the levels of glucokinase and Glucose-6-phosphate, thereby promoting glycogen synthesis. It was reported that DNJ enhanced adiponectin secretion by regulating FOXO1 gene expression, thereby improving insulin sensitivity in mice [22]. A 50 mg/mL concentration of DNJ reduced glucagon-induced glycogenolysis in the rat liver by 75% and decreased hepatic glycogenesis by 60%. Its antiglycolytic properties may be related to the inhibition of the glycogen debranching enzyme activity of DNJ, specifically alpha-1,6-glucosidase [20] (Table 2).

Overall, DNJ’s multifaceted actions, from directly inhibiting intestinal glucose absorption and enhancing cellular uptake via insulin signalling pathways to modulating key enzymes in glycolysis, gluconeogenesis, and the pentose phosphate pathway, underscore its comprehensive approach to restoring glucose homeostasis in T2DM. The integrated regulatory capacity of DNJ has established it as a promising agent for managing hyperglycaemia through distinct yet interconnected mechanisms. Further studies on the effect of DNJ on mitochondrial energy metabolism, including oxidative phosphorylation efficiency, ATP production, and mitochondrial biogenesis, are essential to uncover the underlying mechanism and explore the broader role of glucose and energy homeostasis. In addition to glucose metabolism, lipid metabolism has a direct effect on glucose homeostasis and IR. Consequently, DNJ-mediated regulation of lipid metabolism is critical for effective hyperglycaemia management.

## 4. Lipid Metabolism Disorders in T2DM and the Regulatory Role of DNJ

Adipose tissues can be categorised into three distinct types: WAT, brown adipose tissue (BAT), and beige adipose tissue. WAT stores energy, whereas BAT and beige adipose tissue are responsible for energy generation during thermogenesis. Collectively, these tissues maintain energy balance [58,59]. Adipose tissues play a crucial role in glucose homeostasis by secreting adipokines that influence it [60,61].

Adipose tissue plays a critical role in energy balance. For instance, WAT secretes leptin, an adipokine, that interacts with insulin to maintain blood glucose levels. However, in the case of T2DM, adipose tissues will undergo significant dysfunction in T2DM. Initially, under conditions of excess nutrients or metabolic stress, IR develops, diminishing insulin’s inhibitory effect on lipolysis. This leads to elevated free fatty acid (FFA) levels in the circulation. These FFAs were esterified with glycerol in adipocytes to form triglycerides (TGs), which accumulate in adipocytes and other tissues like the liver and muscle [62]. In the liver, FFAs are further metabolised to produce hepatic acetyl-CoA. This activates PC to drive endogenous glucose production during fasting [63,64]. In addition, this dysfunction has been shown to promote non-insulin-independent gluconeogenesis and lipogenesis, which ultimately drive ectopic lipid deposition [61].

Simultaneously, fatty acid oxidation is impaired in the skeletal muscle. This elevates the level of FFAs, which hinders GLUT4 translocation and glycogen synthase activity [65]. Chronic elevation of FFAs directly impairs pancreatic *β*-cell function, contributing to defects in insulin secretion. Furthermore, in the relatively hypoxic microenvironment of adipose tissue, saturated fatty acids have been observed to activate adenine nucleotide translocase-2 on the inner mitochondrial membrane, thereby further upregulating the expression of hypoxia-inducible factor-1α and resulting in adipocyte dysfunction [66].

**Figure 2:**
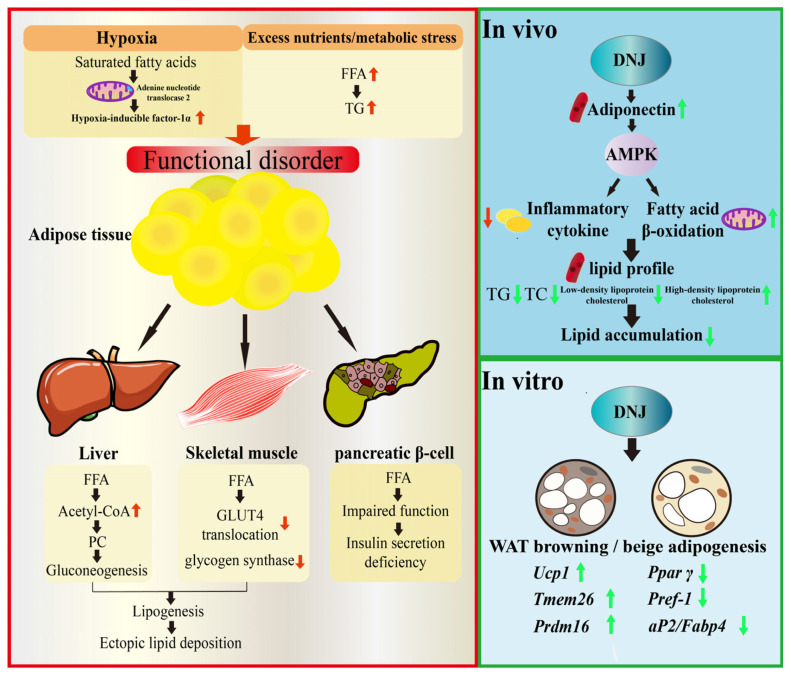
The regulation of DNJ on lipid metabolism disorders in T2DM. The primary function of DNJ is to enhance the metabolic processes of lipids associated with T2DM by regulating plasma lipid profiles, adipocyte browning/beiging, and lipogenic factors reduction. Note: Black arrows indicate upstream and downstream cascade reactions; red arrows indicate that under lipid metabolism disorder conditions, the expression levels of related proteins increase or decrease; green arrows indicate that after DNJ regulates lipid metabolism, the expression levels of related proteins increase or decrease.

DNJ has demonstrated multifaceted effects on lipid metabolism. DNJ can modulate the activity of key enzymes involved in fatty acid metabolism [29] and increase the levels of metabolic activators such as plasma adiponectin. Adiponectin is an adipokine known to activate AMPK, a master regulator of energy metabolism, thereby activating the AMPK signalling pathway and thus upregulating fatty acid *β*-oxidation. This will significantly reduce lipid accumulation and plasma TG levels [67] (Figure 2). Beyond direct enzyme modulation, DNJ also contributes to a healthier lipid profile by inhibiting inflammatory cytokines and reducing lipid peroxidation in skeletal muscle [12].

In addition, DNJ has been shown to upregulate the expression of thermogenesis-related genes, such as uncoupling protein 1 (*Ucp1*), transmembrane protein 26 (*Tmem26*), and PR domain zinc finger protein 16 (*Prdm16*). Simultaneously, DNJ inhibit the expression of key adipogenic transcription factors, including peroxisome proliferator-activated receptor gamma (*Ppar γ*), preadipocyte factor-1 (*Pref-1*), and adipocyte protein 2 (*aP2*)/fatty acid-binding protein 4 (*Fabp4*). This comprehensive regulatory effect has been demonstrated to effectively suppress adipogenesis, enhance thermogenesis, and reduce lipid storage in the 3T3-L1 preadipocyte model [68]. During in vivo studies, DNJ has been shown to beneficially regulate the plasma lipid profile and alleviate HFD-induced dyslipidaemia [69] (Figure 2).

From the above discussion, DNJ improves obesity and related metabolic dysfunction by regulating the secretion of adipokines, the activity of lipid-metabolising enzymes, and key signalling pathways (such as the AMPK/PPAR-γ/UCP1 axis). Further investigation of the impact of DNJ on thermogenic capacity, mitochondrial dynamics, fatty acid oxidation, glucose oxidation, and insulin sensitivity will not only supplement the current understanding of adipose tissue metabolic plasticity but also provide potential therapeutic strategies for T2DM and related metabolic diseases. Beyond glucose and lipid metabolism, IR is a core driver of T2DM that is deeply interconnected with both pathways. DNJ acts holistically to enhance insulin sensitivity and alleviate IR, thereby targeting the root cause of metabolic dysregulation.

## 5. IR in T2DM and the Regulatory Effects of DNJ

### 5.1. The Mechanisms of IR Occurrence

IR plays a vital role in most individuals with T2DM [70]. The development of IR is a multifaceted process driven by a vicious cycle involving chronic inflammation, accumulation of lipotoxic metabolites, mitochondrial dysfunction and oxidative stress, each exacerbating the others. These mechanisms collectively affect organs, such as the liver, muscles, and adipose tissue, leading to impaired signalling pathways and disrupted glucose and lipid metabolism. In the early stages of T2DM, metabolic characteristics include reduced glycogen synthesis and increased IR in the muscles and skeletal muscles owing to lipid accumulation. Typical clinical manifestations of IR include decreased insulin secretion capacity of pancreatic *β*-cells and impaired insulin action, primarily characterised by adipose tissue hypertrophy caused by excess nutrition and ectopic TG deposition in the liver and muscles [61].

First, extensive research has focused on the role of inflammation in IR. While factors like tumour necrosis factor-α (TNF-α) and interleukin-6 (IL-6) are established promoters of IR, the precise initial triggers often stem from chronic metabolic overload, which then propagates these inflammatory pathways [71]. For example, HFD can rapidly activate key inflammatory mediators such as c-Jun N-terminal kinase (JNK) and inhibitory protein (inhibitor of kappa B) kinase. The activation of these mediators is associated with binge eating behaviour and significant weight gain, ultimately leading to the development of IR [72]. Similarly, in the early stages of IR, non-alcoholic steatohepatitis is accompanied by loss of mitochondrial functional flexibility in the liver [73]. Conversely, at the adipose tissue level, IR in adipocytes triggers a local inflammatory response that promotes lipolysis, leading to elevated plasma FFA levels and potentially resulting in pancreatic *β*-cell dysfunction [74]. Additionally, saturated fat intake induces IR in the liver, skeletal muscles, and WAT [75].

IR is closely associated with specific lipotoxicity-related metabolic molecules and their signalling pathways. DAG levels are important predictors of IR. IR is strongly linked to the DAG-protein kinase C (PKC) signalling pathway in obese individuals with non-alcoholic fatty liver disease. Substantial accumulation of DAG within diseased hepatocytes specifically activates PKCε, which translocates to the plasma membrane and disrupts IR tyrosine kinase activity. This inhibits the initiation and transduction of insulin-regulated PI3K-AKT signalling, manifesting as persistent hyperglycaemia along with reduced glycogen synthesis and glucose uptake. Similarly, impaired mitochondrial function in muscle cells leads to the accumulation of sn-1,2-DAG, which induces oxidative stress. These factors collectively increase the levels of mitochondrial reactive oxygen species (ROS) and ceramide, which activate PKCθ and PKCε. These kinases subsequently inhibit the PI3K-AKT pathway through the mechanisms mentioned above, resulting in persistent hyperglycaemia [76,77].

In addition to lipid toxin accumulations, mitochondrial dysfunction and oxidative stress are core mechanisms of IR [78]. Key genes such as peroxisome proliferator-activated receptor gamma coactivator-1α (*Pgc1α*), nuclear respiratory factor 1, and *Ppar γ*, play crucial roles in regulating mitochondrial biogenesis. The downregulation of these genes may impair mitochondrial biogenesis. This is accompanied by the dysregulation of mitochondrial fusion and fission, resulting in excessive mitochondrial fission. Dysfunctional mitochondria promote fatty acid *β*-oxidation and increase the production of ROS [79]. Excessive ROS acts as a key signalling molecule and activates stress kinase pathways, such as JNK and p38 mitogen-activated protein kinase (MAPK) [80]. These kinases also negatively regulate the PI3K-AKT signalling axis by promoting serine phosphorylation of IRS, leading to disrupted fatty acid metabolism and subsequent induction of IR [81].

### 5.2. The Role of DNJ in Improving IR

**Figure 3:**
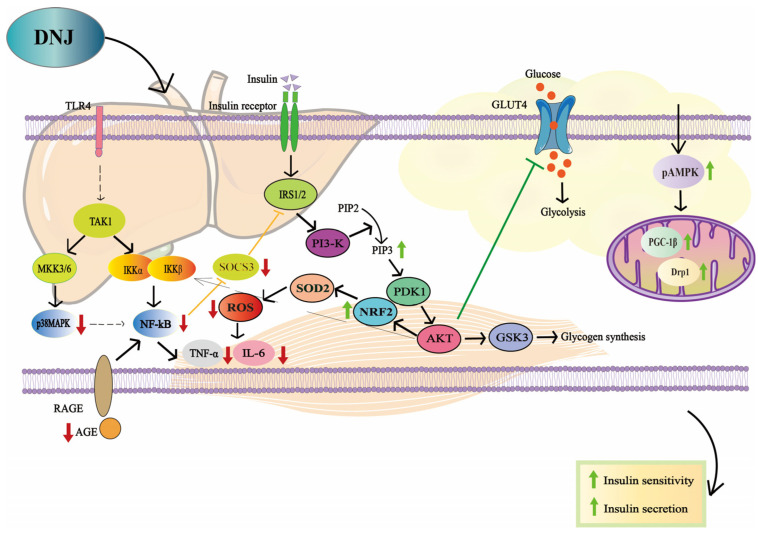
The effect of DNJ on improving IR in T2DM. DNJ exerts its primary ameliorating effects on IR by enhancing insulin sensitivity and secretion capacity in patients with T2DM, through the regulation of signalling pathways such as PI3K-AKT, TLR4/NF-κB, and p38MAPK/NF-κB. Note 1: The definitions of the abbreviations used in the figure are transforming growth factor β-activated kinase 1 (TAK1), mitogen-activated protein kinase kinase 3/6 (MKK3/6), inhibitor of nuclear factor kappa B kinase α/β (IKKα/β), suppressor of cytokine signalling 3 (SOCS3), phosphatidylinositol 4,5-bisphosphate (PIP2), phosphatidylinositol 3,4,5-trisphosphate (PIP3), superoxide Dismutase 2 (SOD2), 3-phosphoinositide-dependent protein kinase 1 (PDK1), glycogen synthase kinase 3 (GSK3), dynamin-related protein 1 (Drp1). Note 2: Black arrows indicate upstream and downstream cascade reactions; red arrows indicate a decrease in protein expression levels; green arrows indicate an increase in protein expression levels.

DNJ can improve insulin sensitivity by upregulating the expression of genes related to the PI3K/AKT signalling pathway, thereby enhancing downstream insulin signalling components [18,47,82] (Figure 3). Toll-like receptors (TLRs), particularly TLR2 and TLR4, can enhance the expression of TNF-α and IL-6 through the nuclear factor kappa B (NF-κB) signalling pathway, thereby promoting the development of IR [83]. DNJ can reduce the expression of TNF-α, IL-6, and suppressor of cytokine signalling 3 by inhibiting the activity of the NF-κB pathway and regulating the TLR4/NF-κB signalling pathway. Through these mechanisms, DNJ has demonstrated potential efficacy in ameliorating IR.

DNJ promotes the expression of *Ppar γ*, CCAAT/enhancer-binding protein α (*C/EBP α*), and the adipocyte differentiation transcription factor sterol regulatory element-binding protein-1 in 3T3-L1 cells. Additionally, DNJ inhibits damage and apoptosis induced by advanced glycation end-products (AGEs) in intestinal endocrine GLUTag cells. It reduces the expression of AGEs and the receptor for AGEs (RAGE), as well as proteins associated with the p38MAPK/NF-κB signalling pathway, thereby improving IR [84]. DNJ can also ameliorate IR by reducing circulating plasma endotoxin levels [85].

DNJ activates the AMPK signalling pathway in liver tissue, increasing its phosphorylation level (p-AMPK). This upregulates the expression of PGC-1β, promoting mitochondrial biogenesis and enhancing fatty acid *β*-oxidation capacity [86]. DNJ can also increase the expression of dynamin-related protein 1 in mitochondria of peripheral blood mononuclear cells, regulating mitochondrial dynamics and maintaining energy homeostasis [12], effectively alleviating hepatic lipid accumulation and IR. In addition, DNJ upregulates the protein expression of NRF2, activating the transcription of downstream antioxidant genes such as superoxide dismutase 2, efficiently clearing excess ROS, and reducing the production of pro-inflammatory cytokines (such as TNF-α and IL-6), breaking the vicious cycle between inflammation and oxidative stress and improving IR [12,87].

Collectively, these actions of DNJ, ranging from direct modulation of insulin signalling components to mitigating inflammation and reducing lipotoxic insults, converge to restore cellular responsiveness to insulin and alleviate the multifaceted pathology of IR. Despite its potential, the pharmacological impact of DNJ on the transition from prediabetes to T2DM and on insulin sensitivity in established T2DM has scarcely been explored, highlighting the need for rigorous clinical and mechanistic studies.

Furthermore, IR is strongly influenced by the gut microenvironment. Dysbiosis can exacerbate IR through systemic inflammation driven by bacterial endotoxins, while the resulting hyperglycaemic and lipotoxic states further compromise gut barrier function. This bidirectional relationship highlights the gut microbiota as a critical therapeutic target. DNJ has been shown to regulate this axis, suggesting that its antidiabetic effects are partly mediated through microbiota modulation, as discussed in the following section.

## 6. The Impact of T2DM on Intestinal Microbiota (IM) and the Regulatory Role of DNJ

### 6.1. Changes in IM

The IM regulates blood glucose levels and influences satiety through complex neural networks, peptide hormones, and endocrine hormone release mechanisms [88,89]. In T2DM patients, the composition of the IM is significantly altered, typically characterised by a decrease in beneficial bacteria and an increase in potentially harmful species. For instance, beneficial microbes like *Allobaculum* and *Bifidobacterium* are known to reduce endotoxin levels and inflammation, contributing to metabolic and immune regulation [90,91]; T2DM patients often exhibit a higher abundance of Gram-negative bacteria, particularly *Proteobacteria* [92]. Among these bacteria, endotoxins produced by *Shigella* can translocate and form complexes with lipopolysaccharide (LPS)-binding proteins and TLR4, triggering a series of inflammatory responses through the NF-κB signalling pathway, thereby exacerbating endotoxemia [93]. Additionally, D-lactate, produced by intestinal bacterial fermentation, enters the bloodstream in greater amounts when intestinal permeability increases [94]. IM can decompose tryptophan metabolites through the indole, kynurenine, and serotonin pathways or break down histidine into imidazole propionate. Alterations in these metabolic pathways play a pivotal role in the development of IR [95,96].

IM disorders may also reduce the production of beneficial metabolites, such as short-chain fatty acids, trimethylamine, and branched-chain amino acids, thereby affecting glucose homeostasis by altering the energy balance and bioavailability of amino acids, blocking liver insulin signalling pathways, and exacerbating inflammation [70,97,98,99]. HFD has been shown to promote gut dysbiosis by enriching pro-inflammatory pathogenic bacterial taxa, thereby increasing the abundance and translocation of LPS, the primary component of the outer membrane of Gram-negative bacteria, into systemic circulation. This elevation in circulating LPS activates TLR4 signalling, which in turn triggers chronic low-grade inflammation and contributes to the development of IR. Additionally, HFD enhances the intestinal absorption of dietary fatty acids, further exacerbating systemic lipid metabolism disturbances and promoting dyslipidaemia [100,101]. These findings underscore the critical role of the IM in the pathogenesis of T2DM and identify promising molecular and microbial targets for the development of novel therapeutic interventions.

### 6.2. The Regulatory Effect of DNJ on the IM

DNJ possesses the ability to modulate the composition of the IM, enhancing the abundance of certain beneficial microorganisms, such as *Akkermansia*, *Clostridium* group XIVa, *Butyricicoccus*, *Bifidobacterium*, and *Lachnospiraceae* NK4A136. Additionally, it reduces the abundance of potentially harmful microbes, including *Ruminococcaceae*, *Faecalibaculum*, *Weissella*, specific groups within *Lachnospiraceae* (*Lachnospiraceae_UCG-006*), *Clostridium sensu_stricto_1*, *Prevotellaceae* Ga6A1 group, *Enterorhabdus*, *Blautia*, *Anaerostipes*, and *Klebsiella* [21,25,26,102].

These microbial communities exert a direct influence on body physiology through their metabolic products. It demonstrated that the presence of beneficial bacteria, such as *Akkermansia*, which produce acetate, and *Bifidobacterium*, which produce lactate and acetate, in addition to several species within the *Clostridium* group XIVa, *Lachnospiraceae* NK4A136, and *Butyricicoccus*, which produce butyrate, contributes to the maintenance of intestinal barrier integrity. Furthermore, these bacteria reduced the expression of pro-inflammatory cytokines, improved lipid profiles, and consequently enhanced glucose tolerance and insulin sensitivity [103]. Conversely, a decline in potentially deleterious bacteria, including *Faecalibaculum*, *Lachnospiraceae_UCG-006*, *Clostridium sensu stricto 1*, *Enterorhabdus*, *Blautia*, and *Klebsiella*, exacerbates the expression of pro-inflammatory cytokines, compromises lipid profile abnormalities, and compromises the integrity of the intestinal barrier. Changes in the abundance of *Weissella* and *Prevotellaceae* Ga6A1 group have a significant impact on the expression of gluconeogenic enzymes, with a concomitant decrease in the expression of glycolytic enzymes. These changes impair glucose metabolism and lead to the development of T2DM [12,104].

**Figure 4:**
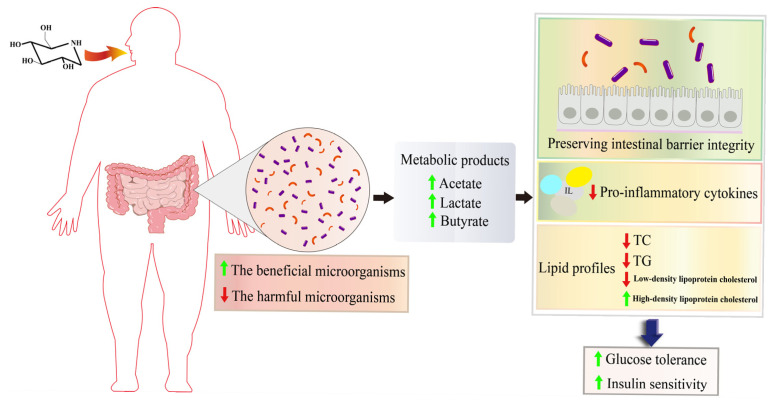
Mechanism of DNJ in modulating gut microbiota in T2DM. DNJ alleviates T2DM-associated metabolic disorders by enriching beneficial bacteria (e.g., *Akkermansia*) and suppressing pathogenic bacteria (e.g., *Klebsiella*), thereby maintaining microbial homeostasis and enhancing intestinal barrier function. Note: Black arrows indicate upstream and downstream cascade reactions; red arrows indicate a decrease in protein expression levels; green arrows indicate an increase in protein expression levels.

DNJ enhances the gut barrier, reduces the levels of inflammatory factors, and improves lipid metabolism and IR by remodelling IM, thereby reducing the impact of T2DM and its complications [23,85] (Figure 4). Although the regulatory impact of DNJ on IM has been substantiated, its specific effects on IR and its underlying mechanisms remain unclear. Further research is necessary to determine the potential benefits of DNJ in ameliorating IR.

## 7. Research on the Potential Mechanisms of DNJ in T2DM Based on Network Pharmacology

Network pharmacology is an integrative approach that combines systems biology, bioinformatics, and multi-omics technologies to elucidate complex interactions between bioactive compounds and their molecular targets within biological networks. Unlike conventional pharmacological models, which typically focus on a single target, network pharmacology enables the systematic prediction and analysis of multi-target effects of individual or compound mixtures, thereby offering a more holistic understanding of drug action [105,106,107]. This approach has been extensively employed to elucidate the molecular mechanisms underlying the therapeutic effects of traditional Chinese medicine (TCM), offering a system-level framework to identify synergistic interactions among multi-component formulations against diseases [105,107]. As DNJ is a natural compound present in botanicals such as mulberry leaves, which are often used in TCM, network pharmacology offers a powerful tool to systematically investigate its multi-target actions in T2DM. Numerous researchers have integrated network pharmacology to study the health benefits of mulberry leaves and DNJ in diabetes [10,108,109].

Through network pharmacology analysis, Lv et al. [109] revealed that the active components in mulberry leaves improve blood glucose and IR mainly through the PI3K-AKT, lipid, and AGE-RAGE signalling pathways. Moreover, maltase-glucoamylase, sucrase-isomaltase proteins, and α-galactosidase are the core targets of mulberry leaf alkaloids (DNJ), which improve IR by regulating glucose metabolism. In addition, network pharmacology predicted and analysed the protective mechanism of DNJ on pancreatic β-cells, involving carbohydrate metabolism, AMPK, MAPK, and PPAR signalling pathways, which have the potential to improve T2DM characteristics [10].

In the intricate regulatory network of T2DM, DNJ exhibits multi-target and multi-pathway synergistic intervention features. Network pharmacology analysis revealed that mulberry leaf extract modulates lipid and glucose metabolism through the lipid metabolism, atherosclerosis, PI3K–AKT, and AGE–RAGE signalling pathways, and exerts anti-inflammatory effects by targeting cytokines such as IL-6, IL-10, and TNF [82,110]. Furthermore, molecular docking results revealed that DNJ exhibits strong binding affinity for key diabetes-related target proteins, insulin-related factors, Ppar γ, PI3K, GLUT4, and IL-6, indicating stable ligand–receptor complex formation [82]. Li et al. [10] employed network pharmacology to analyse the effects of DNJ on nine diabetes-related target genes. The authors further reported that DNJ exerts a protective effect against high glucose–induced *β*-cell damage by upregulating CCAAT/enhancer-binding protein α, a key molecular target of DNJ. The primary signalling pathways include AMPK, MAPK, and PPAR, which are associated with enhanced insulin signalling.

The application of DNJ is broad, ranging from dietary interventions to precise targeted therapeutic exploration. At present, the DNJ as a functional food and dietary supplement is clearly delineated, and market applications are well-established. DNJ in mulberry leaf tea and mulberry leaf powder has been shown to inhibit postprandial blood glucose increase in healthy individuals and those with impaired glucose tolerance when consumed as a beverage. The utilisation of silkworm pupae powder and silkworm excrement extracts, as high-protein raw materials enriched with DNJ, has been employed in the development of specialised dietary foods. Furthermore, the employment of microbial fermentation technology (for example, *Bacillus subtilis* fermenting soybean dregs) to ferment food matrices facilitates the large-scale production of food-grade DNJ functional products. In the context of dietary supplements and nutritional health products, DNJ is formulated in standardised forms (e.g., capsules, tablets, and concentrated powders) and is used in combination with components such as mulberry leaf polysaccharides and flavonoids. This exerts a synergistic effect, assisting in the management of individuals with prediabetes and early-stage T2DM, while providing nutritional support for healthy populations. About therapeutic and pharmacological applications, DNJ has been demonstrated at both the cellular and animal levels to act through multi-target and multi-pathway regulation, thus offering preventive and therapeutic effects for T2DM and its complications [111,112].

Although network pharmacology has identified core DNJ targets in T2DM regulation (such as the PI3K-AKT and AMPK signalling pathways), existing studies are mostly limited to static analyses of single compounds or crude extracts, overlooking the complex practical variables. For example, food processing methods (heat treatment, fermentation) may alter the bioavailability of DNJ; dietary composition and individual factors (degree of obesity, intestinal microbiota composition) may influence the absorption and metabolic kinetics. In addition, target selection strategies differ between functional foods, which prioritise prevention and metabolic homeostasis in at-risk populations, and therapeutic contexts, which focus on intervening in the pathological process for diagnosed patients. This difference may result in shifts in predicted action targets and related signalling pathways in network pharmacology. Future network pharmacology research should integrate these multidimensional parameters to build predictive models that more closely reflect real dietary scenarios, ensuring the scientific validity and practical applicability of DNJ in functional foods.

## 8. The Bioavailability, Long-Term Safety, and Pharmacokinetics of DNJ

### 8.1. Bioavailability of DNJ

The bioavailability of DNJ should be considered when developing it for therapeutic or functional food applications. It has been observed that when DNJ is administered orally at 80 mg/kg, the oral bioavailability is approximately 50 ± 9%, which is attributed to the good absorption capacity of DNJ in the intestines [113]. Following oral administration at 100 mg/kg, DNJ demonstrated rapid absorption (*T*_max_ = 30 min) with a C_max_ of 1500 ng/mL and a short elimination half-life [85]. Despite better α-glucosidase inhibition properties of DNJ compared to synthetic analogues like miglitol and voglibose, its bioavailability is less than that of them. Further, DNJ is unstable in vivo [40]. However, to improve the bioavailability and stability of DNJ, researchers are optimising and standardising DNJ extraction methods [114], modifying DNJ’s structure [40], optimising DNJ administration methods (combined with bioenhancers), adjuvant formulations (encapsulation, nano-formulations), and targeted delivery systems [85], aiming to increase DNJ’s potential in improving T2DM from multiple angles.

### 8.2. Safety Considerations for Chronic DNJ Use

Compared with drugs for the treatment of T2DM, reports of side effects from DNJ are fewer, and it is relatively safer. Therefore, some researchers have suggested the long-term use of DNJ to manage T2DM [114,115,116]. Studies have found that oral DNJ at 6–12 mg/d is safe, and approximately 6.3 mg of DNJ can effectively reduce postprandial blood glucose levels in humans [85]. However, most current safety studies focus on mulberry leaf extracts containing different doses of DNJ, and there are few long-term safety studies on pure DNJ, with no standardised dosage guidelines. It is necessary to evaluate the long-term safety of DNJ from the perspectives of different doses, administration methods, duration, individual differences, and allergic reactions, to provide an effective and safe dosage range for the prevention and treatment of T2DM with DNJ.

### 8.3. Pharmacokinetics of DNJ

Pharmacokinetic studies of DNJ guide the prevention and treatment of T2DM. Research has found that the level of DNJ in rats’ plasma is dose-dependent [117]. After oral administration of DNJ (80 mg/kg) to rats, the elimination half-life in the body is relatively short, approximately 2.5 h [113]. Moreover, DNJ is rapidly absorbed in the body, with a *T*_max_ of approximately 0.5 h, making it suitable for postprandial blood glucose control [85,113]. In addition, after oral administration, DNJ is widely distributed to tissues such as the liver, kidney, spleen, pancreas, small intestine, and large intestine, but mainly distributes in organ tissues, with an apparent volume of distribution reaching 7.45 L/kg. After intravenous injection, DNJ is mainly distributed in the blood, with an apparent volume of distribution of 0.98 L/kg [85,113]. However, pharmacokinetic studies of DNJ are not yet in-depth and need to be further elucidated through research on optimising DNJ administration methods, clinical trials of pure DNJ, and synergistic effects with other bioactive substances to facilitate the long-term, stable, and effective prevention and treatment of T2DM.

Although DNJ has considerable application potential, improving its bioavailability in the body, determining its long-term safety, clinical efficacy, range of administration, and enhancing tissue targeting are also among the current key research directions for the prevention and treatment of T2DM.

## 9. Conclusions

This review integratively analyses the synergistic regulatory mechanisms of DNJ on the core pathophysiological metabolism of T2DM. We systematically elucidated how DNJ, a promising natural bioactive compound with minimal side effects, intervenes in critical metabolic pathways to manage T2DM. DNJ demonstrates multifaceted therapeutic potential, including inhibition of glucose absorption, enhancement of glucose metabolism, mitigation of lipid toxicity, improvement of IR, and modulation of the gut microbiota. Although a few studies on network pharmacology have explained the multiple targets of DNJ in diabetes management, comprehensive studies remain limited.

Nevertheless, the therapeutic potential of DNJ in T2DM requires deeper exploration in three key areas: (1) adipose tissue metabolic plasticity: the effects of DNJ on the browning of WAT, activation of BAT, and the secretion profile of adipokines, as well as its specific intervention targets in obesity-related T2DM; (2) islet cell protection and functional regulation: the key molecular targets and signalling pathways involved in DNJ’s dual regulation of pancreatic α-cell glucagon secretion and β-cell insulin synthesis/secretion; (3) mitochondrial quality control: the regulatory mechanisms of DNJ on tissue-specific mitochondrial biogenesis, dynamics (fusion/fission balance), and mitophagy, and its effects on energy metabolism. Additionally, systematic studies are needed to overcome the limitations of low oral bioavailability and poor tissue-specific distribution. This includes formulation optimisation (such as nanocarriers or liposome encapsulation) to improve stability and intestinal absorption, elucidating metabolic characteristics at the in vivo level, and clarifying effective doses, safety, and drug interactions in clinical settings. This research will facilitate the development of DNJ as a core component of functional foods or dietary supplements for the prevention and treatment of T2DM, providing a basis for its scientific application.

In conclusion, DNJ is a promising natural compound for T2DM management, offering a multi-targeted approach to treat this complex metabolic disorder. Further research and validation are needed to confirm the efficacy of DNJ as a strategy for the prevention and treatment of T2DM, particularly in its early stages.

## Figures and Tables

**Table 1:** Research from in vivo on DNJ for T2DM management.

Dosage	Purity	Duration	Method of Giving	Research Subject	Results	Ref.
40, 80 mg/kg BW/D	HPLC ≥ 95% (derived from mulberry leaf extract)	4 weeks	Tail vein injection	Male wild-type C57 BLKS mice and C57 BLKS/Leprdb (db/db) mice	DNJ improves insulin sensitivity in db/db mice by activating the insulin signalling phosphatidylinositol 3-kinase (PI3K)-protein kinase B (AKT) pathway in skeletal muscle, thereby alleviating hyperglycaemia.	[18]
40, 80 mg/kg BW/D	HPLC ≥ 95% (derived from mulberry leaf extract)	4 weeks	Tail vein injection	Male wild-type C57 BLKS mice and C57 BLKS/Leprdb (db/db) mice	DNJ can improve hepatic insulin sensitivity in db/db mice by enhancing the insulin-stimulated protein kinase B/glycogen synthase kinase-3β signalling pathway and regulating glucose metabolism enzymes. DNJ can also improve lipid balance in db/db mice and alleviate hepatic steatosis.	[19]
50 mg/kg BW, twice a day.	HPLC ≥ 95% (derived from mulberry leaf extract)	3 days	Gastric injection	Male ICR mice	DNJ inhibits glucose absorption in the small intestine by reducing the expression of proteins involved in the epithelial glucose transport system, and maintains stable blood sugar levels by directly regulating the expression of enzyme proteins involved in hepatic glycolysis and gluconeogenesis.	[20]
62.5, 125 mg/kg BW/D	HPLC ≥ 95% (derived from mulberry leaf extract fermented by microbial strains)	4 weeks	Oral gavage	Male Kunming mice	DNJ significantly lowers blood glucose and insulin levels in diabetic mice, improves blood lipid profiles and IR by increasing the protein expression of key glycolytic enzymes, decreasing the protein expression of key gluconeogenic enzymes, upregulating liver-related protein expression to activate the insulin signalling pathway, and reshaping the disrupted gut microbiota.	[21]
10 mg/kg BW/D	HPLC ≥ 95% (derived from *Bacillus* amyloliquefaciens AS 385 cultured broth powder)	10 weeks	Feeding	C57BL/6J mice	DNJ can improve obesity, impaired glucose tolerance, and reduced insulin sensitivity caused by a high-fat diet (HFD) by regulating the expression of genes involved in insulin signalling and lipid metabolism, such as adiponectin in white adipose tissue (WAT).	[22]
20 mg/kg BW/D	HPLC ≥ 98%	10 weeks	Feeding	Male C57 BL/6 mice	DNJ improves blood glucose homeostasis and lipid metabolism in prediabetic mouse models induced by HFD and streptozotocin by protecting the integrity of the intestinal barrier and inhibiting the LPS/TLR4/NF-κB axis, reducing levels of IL-6, TNF-α, and LPS associated with IR.	[23]
20 mg/kg BW/D	HPLC ≥ 98% (derived from mulberry leaf extract)	9 weeks	Oral gavage	Male mice [BKSCg-Dock7m^+^/m^+^ Leprdb/Nju (db/db)] and C57 BLKS/J Nju (db/m) mice	DNJ enhances the entry of glucose into the TCA cycle and lowers the urinary levels of amino acid and cholesterol (TC) metabolites, restoring normalcy to metabolic disorders such as glucose metabolism, energy metabolism, and lipid metabolism.	[24]
0.1 mg/mL	DNJ Pure Product	4 months	Drinking water feeding	Male C57BL/6J mice	DNJ alleviates hepatic steatosis and systemic chronic inflammation by regulating impaired glucose tolerance and hyperlipidaemia, and mitigates the progression of HFD-induced non-alcoholic steatohepatitis by reconstructing the gut microbiota composition.	[25]
50 mg/kg BW/D	HPLC ≥ 95% (derived from mulberry leaf extract)	12 weeks	Oral gavage	Male and female ICR mice	DNJ improves hypercholesterolemia induced by streptozotocin and HFD in female mice by regulating gut microbiota and downregulating key genes involved in fat and TC biosynthesis.	[26]
50 mg/kg BW/D	HPLC ≥ 95% (derived from mulberry leaf extract)	12 weeks	Oral gavage	Male and female C57BM/6J mice	DNJ inhibits HFD-induced hyperlipidaemia and modulates the gut microbiota in a sex-specific manner, particularly increasing *Akkermansia*, revealing a new characteristic of DNJ’s pharmacological effects and providing new insights into its mechanism for alleviating hypertension.	[27]
1 mg/kg BW/D	HPLC ≥ 95% (derived from mulberry leaf extract)	4 weeks	Gastric tube feeding	Male Sprague-Dawley rats	DNJ increases plasma adiponectin levels, enhances the expression of AMP-activated protein kinase (AMPK) mRNA, activates the *β*-oxidation system, inhibits lipid accumulation in the liver, and improves hepatic oxidative stress.	[28]
5 mg/kg BW/D	HILIC ≥ 98% (derived from mulberry leaf extract)	12 weeks	Gastric tube feeding	C57BM/6J mice	DNJ can promote the increase in plasma adiponectin levels and activate the *β*-oxidation system, inhibiting lipid accumulation in the liver and reducing plasma triglycerides, thereby preventing diet-induced obesity.	[29]
1 μL DNJ (50 μg/mL)	DNJ pure product	6 h	Intracerebroventricular injection	Male C57BL/6J mice	DNJ can reduce food intake and obesity in rats by lowering hypothalamic endoplasmic reticulum stress and activating the leptin-induced janus kinase 2/signal transducer and activator of transcription 3 signalling pathway.	[30]
5, 25 mg/kg BW/D, five days a week.	Mulberry leaf ethanol extract: DNJ (3.75%) and resveratrol (0.015%)	12 weeks	Oral	C57BM/6J mice	Mulberry leaf extract containing DNJ can reduce liver fat accumulation, fibrosis, and oxidative stress in mice fed a HFD induced by obesity.	[31]
DNJ (50 mg/kg BW/D) + polysaccharide (100 mg/kg BW/D)	HPLC ≥ 95% (derived from mulberry leaf extract)	90 days	Oral gavage	Male ICR mice	DNJ–polysaccharide complexes inhibit glucose absorption in the small intestine by reducing the expression of glucose transporters across the epithelium, maintain stable blood sugar levels by directly modulating the expression of enzymes involved in glycolysis and gluconeogenesis in the liver, and restore the damaged pancreas to normal by scavenging free radicals and promoting *β*-cell proliferation.	[32]
DNJ (50 mg/kg BW/D) + polysaccharide (100 mg/kg BW/D)	DNJ LC-MS ≥ 95%, polysaccharide HPLC ≥ 95%	12 weeks	Oral gavage	Male ICR mice	DNJ–polysaccharide mixture alleviates postprandial hyperglycaemia, reduces the toxic effects of persistent supraphysiological glucose on pancreatic *β*-cells, and repairs damaged islet *β*-cells by activating the pancreatic-duodenal homeobox 1/insulin-1 signalling pathway, glucose kinase, phosphoenolpyruvate carboxykinase, and glucose-6-phosphatase, as well as scavenging free radicals.	[33]
(DNJ: flavonoid compounds: polysaccharides = 1:6:8) 100 mg/kg BW/D	Mulberry leaf extract: polysaccharides, flavonoids, and DNJ are 32.60%, 52.34%, and 70.40%, respectively.	6 weeks	Oral gavage	Male Sprague-Dawley rats	Multiple components from mulberries can alleviate inflammation and oxidative damage in T2DM rats by regulating the PI3K/Akt signalling pathway, improve hepatic glucose and lipid metabolism disorders, and reduce IR, providing a new perspective for research on the multi-component, multi-target hypoglycaemic effects of mulberries.	[34]
DNJ (2 mg/kg BW/D) and baicalein (25 mg/kg BW/D)	≥98%	10 weeks	Feeding	Male Sprague–Dawley rats	The synergistic delivery of natural bioactive substances can lower blood sugar, improve insulin sensitivity, reduce systemic inflammation, decrease fat accumulation, and alleviate organ damage, while also regulating gut microbiota homeostasis and repairing the intestinal barrier.	[35]
DNJ (200 mg/kg BW/D) and theaflavins (100 mg/kg BW/D)	Pure product	10 weeks	Feeding	Male C57BL/6J mice	DNJ and theaflavins alleviate HFD-induced inflammation and IR by targeting prostaglandin-endoperoxidesynthase2/matrix metalloproteinase-9 and regulating the TNFα/AKT/Glycogen Synthase Kinase 3/GLUT2 axis, exerting a synergistic hypoglycaemic effect.	[36]
DNJ (5 mg/kg) + morin (25 mg/kg)	HPLC ≥ 98%	9 weeks	Feeding	Male C57BM/6 mice	Low-dose DNJ combined with morin can improve IR and lipid accumulation by inhibiting the expression of suppressor of cytokine signalling 3, promoting the expression of PPARγ and suppressor of cytokine signalling 2, and inhibiting the signalling of cluster of differentiation 36/sterol regulatory element-binding protein-1/fatty acid synthase, effectively preventing the progression of T2DM by 87.56%.	[37]

**Table 2:** Regulation of key enzymes in glucose metabolic pathways in the body.

Metabolic Pathway	Key Regulatory Enzymes	Allosteric Regulation		Hormonal Regulation		Covalent Modification	
		Inhibitors	Activators	Inhibitors	Activators	Inhibitors	Activators
Glycolysis Pathway	Hexokinase	Glucose-6-phosphate	Mg^2+^	Glucagon	Insulin	Not specified	
	Phosphofructokinase	ATP, Citric acid	AMP, Fructose-2,6- Bisphosphate	Glucagon	Insulin	Phosphorylation	Dephosphorylation
	Pyruvate Kinase	ATP, Alanine	Fructose-1,6- bisphosphate	Glucagon	Insulin	Phosphorylation	Dephosphorylation
Tricarboxylic Acid Cycle	Citrate Synthase	ATP, Succinyl-CoA, NADH	ADP	Insulin (indirect effect)		Not specified	
	Isocitrate Dehydrogenase	ATP, Succinyl-CoA, NADH	ADP, Ca^2+^	Insulin (indirect effect)		Phosphorylation	Dephosphorylation
	α-Ketoglutarate Dehydrogenase Complex	Succinyl-CoA, NADH	Ca^2+^	Insulin (indirect effect)		-	
Pentose Phosphate Pathway	Glucose-6- phosphate Dehydrogenase	NADPH	NADP^+^	Insulin (indirect effect)		Not specified	
Gluconeogenesis Pathway	Fructose-1,6- Bisphosphatase	AMP, Fructose-2,6- Bisphosphate	ADP	Glucagon	Insulin	Phosphorylation	Dephosphorylation
	Pyruvate Carboxylase	ADP	Acetyl-CoA	Insulin	Glucagon	Dephosphorylation	Phosphorylation
	Pyruvate Kinase	ATP, Alanine	Fructose-1,6- Bisphosphate	Glucagon	Insulin	Phosphorylation	Dephosphorylation
Glycogen Synthesis and Degradation	Glycogen Synthase	AMP	Glucose-6- phosphate	Epinephrin-e, Glucagon	Insulin	Phosphorylation	Dephosphorylation
	Phosphorylase	Glucose-6- phosphate, ATP, Low concentration of glucose	AMP, Ca^2+^	Insulin	Epinephrin-e, Glucagon	Dephosphorylation	Phosphorylation

## Data Availability

No new data were created or analyzed in this study. Data sharing is not applicable to this article.

## Abbreviations

The following abbreviations are used in this manuscript:

DNJ	1-deoxynojirimycin
AKT	Protein kinase B
DM	Diabetes mellitus
GLUT2	Glucose transporter 2
T2DM	Type 2 diabetes mellitus
PI3K	Phosphatidylinositol 3-kinase
AMPK	AMP-activated protein kinase
SGLT1	Sodium–glucose cotransporter 1
IR-β	Insulin receptor β
TCA	Tricarboxylic acid
HK	Hexokinase
PFK	Phosphofructokinase
PK	Pyruvate kinase
HFD	High-fat diet
PC	Pyruvate carboxylase
IRS	Insulin receptor substrate
ATPase	Adenosine triphosphatease
NADPH	Nicotinamide adenine dinucleotide phosphate hydrogen
NRF2	Nuclear factor erythroid 2-related factor 2
OGG1	8-Oxoguanine DNA Glycosylase 1
FOXO1	Forkhead box protein O1
UCP1	Uncoupling protein 1
WAT	White adipose tissue
BAT	Brown adipose tissue
FFA	Free fatty acids
TG	Triglycerides
TC	Cholesterol
*Tmem26*	Transmembrane protein 26
*Prdm16*	PR domain zinc finger protein 16
*Pref-1*	Preadipocyte factor-1
*aP2*	Adipocyte protein 2
*Fabp4*	Fatty acid-binding protein 4
IL-6	Interleukin-6
DAG	Diacylglycerol
PKC	Protein kinase C
TLRs	Toll-like receptors
RAGE	Receptor for AGEs
PPAR-γ	Peroxisome proliferator-activated receptor γ
TNF-α	Tumour necrosis factor-α
JNK	C-Jun N-terminal kinase
ROS	Reactive oxygen species
MAPK	Mitogen-activated protein kinase
NF-κB	Nuclear factor kappa B
C/EBP α	CCAAT/enhancer-binding protein α
AGEs	Advanced glycation end-products
TCM	Traditional Chinese medicine
IM	Intestinal microbiota
LPS	Lipopolysaccharide
BW	Bodyweight
LC-MS	Liquid chromatography–mass spectrometry
PGC-1α	Peroxisome proliferator-activated receptor gamma coactivator-1α
IR	Insulin resistance
HPLC	High pressure liquid chromatography
HILIC	Hydrophilic interaction liquid chromatography
